# Supplementary material for: Resolving the full spectrum of human genome variation using Linked-Reads
Source: Genome Res. 2019 Apr;29(4):635–45. doi: 10.1101/gr.234443.118 (PMC6442396; doi:10.1101/gr.234443.118)
Supplement: Supplemental Material [file supp_gr.234443.118_Supplemental_longranger-2.2.zip › longranger-2.2.tar.bz2!/lr2.2-public/lib/stan/cmdstan/stan/doc/api/html/check__unit__vector_8hpp_source.html]

Stan: src/stan/math/error\_handling/matrix/check\_unit\_vector.hpp Source File


|  |  |
| --- | --- |
|  | Stan  2.5.0  probability, sampling & optimization |


- Main Page
- Related Pages
- Namespaces
- Classes
- Files

- File List
- File Members

All Classes Namespaces Files Functions Variables Typedefs Enumerations Enumerator Friends Macros Pages

- src
- stan
- math
- error\_handling
- matrix

check\_unit\_vector.hpp

Go to the documentation of this file.

1 #ifndef STAN\_\_MATH\_\_ERROR\_HANDLING\_\_MATRIX\_\_CHECK\_UNIT\_VECTOR\_HPP

2 #define STAN\_\_MATH\_\_ERROR\_HANDLING\_\_MATRIX\_\_CHECK\_UNIT\_VECTOR\_HPP

3

4 #include <sstream>

5 #include <stan/math/matrix/Eigen.hpp>

6 #include <stan/math/error\_handling/dom\_err.hpp>

7 #include <stan/math/error\_handling/matrix/constraint\_tolerance.hpp>

8

9 namespace stan {

10

11  namespace math {

12

26  template <typename T\_prob, typename T\_result>

27  bool check\_unit\_vector(const char\* function,

28  const Eigen::Matrix<T\_prob,Eigen::Dynamic,1>& theta,

29  const char\* name,

30  T\_result\* result) {

31  if (theta.size() == 0) {

32  std::string message(name);

33  message += " is not a valid unit vector. %1% elements in the vector.";

34  return dom\_err(function,0,name,

35  message.c\_str(),"",

36  result);

37  }

38  T\_prob ssq = theta.squaredNorm();

39  if (!(fabs(1.0 - ssq) <= CONSTRAINT\_TOLERANCE)) {

40  std::stringstream msg;

41  msg << "in function check\_unit\_vector(%1%), ";

42  msg << name << " is not a valid unit vector.";

43  msg << " The sum of the squares of the elements should be 1, but is "

44  << ssq;

45  std::string tmp(msg.str());

46  return dom\_err(function,ssq,name,

47  tmp.c\_str(),"",

48  result);

49  }

50  return true;

51  }

52

53

54  template <typename T>

55  inline bool check\_unit\_vector(const char\* function,

56  const Eigen::Matrix<T,Eigen::Dynamic,1>& theta,

57  const char\* name,

58  T\* result = 0) {

59  return check\_unit\_vector<T,T>(function,theta,name,result);

60  }

61

62

63  }

64 }

65 #endif

dom\_err.hpp

stan::agrad::fabs

fvar< T > fabs(const fvar< T > &x)

**Definition:** fabs.hpp:18

stan::math::dom\_err

bool dom\_err(const char \*function, const T &y, const char \*name, const char \*error\_msg, const T\_msg error\_msg2, T\_result \*result)

**Definition:** dom\_err.hpp:36

constraint\_tolerance.hpp

stan::math::check\_unit\_vector

bool check\_unit\_vector(const char \*function, const Eigen::Matrix< T\_prob, Eigen::Dynamic, 1 > &theta, const char \*name, T\_result \*result)

Return true if the specified vector is unit vector.

**Definition:** check\_unit\_vector.hpp:27

stan::math::CONSTRAINT\_TOLERANCE

const double CONSTRAINT\_TOLERANCE

The tolerance for checking arithmetic bounds In rank and in simplexes.

**Definition:** constraint\_tolerance.hpp:11

Eigen.hpp


---

[ Stan Home Page ]

*© 2011–2014,
Stan Development Team.*
